# Longitudinal observation of subretinal fibrosis in Vogt-Koyanagi-Harada disease

**DOI:** 10.1186/s12886-018-0670-0

**Published:** 2018-01-15

**Authors:** Chan Zhao, Fangtian Dong, Fei Gao, Xinshu Liu, Minghang Pei, Shanshan Jia, Meifen Zhang

**Affiliations:** Ophthalmology Department, Peking Union Medical College Hospital, Chinese Academy of Medical Sciences, 1 Shuaifuyuan, Wangfujing, Dongcheng District, Beijing, 100730 China

**Keywords:** Vogt-Koyanagi-Harada disease, Subretinal fibrosis, Subretinal exudates, Spectral domain optical coherence tomography, Pathogenesis

## Abstract

**Background:**

Subretinal fibrosis (SRF) is a vision-threatening complication of Vogt-Koyanagi-Harada disease (VKH). It has long been recognized as a sequela of chronic inflammation. The developmental process of SRF, however, has not been described. The purpose of this study is to provide longitudinal observations of SRF in VKH.

**Methods:**

Retrospective chart review of 10 VKH patients referred to our group between January 2008 and September 2015 at acute uveitic stage with SRF at presentation or who developed SRF during follow up.

**Results:**

Ten patients (6 males and 4 females) with a median age of 39.0 (range, 23 to 58) years old were included. The median disease duration at presentation and median duration of follow up were 25.5 (range 5 to 60) days and 32.5 (range 13 to 61) months respectively. At presentation, all patients except one had been inappropriately treated with glucocorticosteroid (insufficiently dosed or tapered too fast) for longer than 2 weeks. Despite large dose oral glucocorticosteroid (1 mg/kg/d prednisone or equivalent) with slow tapering in combination with at least one immunomodulatory agent (cyclosporin A, cyclophosphamide or azathioprine) after presentation, all patients developed bilateral SRF within the first 4 months of disease course and 7 patients within the first 2 months. In 8 patients, shape-change/migration and progressive proliferation/pigmentation of SRF was observed over a period of several months after its formation, and then became quiescent but may further underwent depigmentation or pigmentation. SRF involved macula in 12 eyes (7 patients) and caused treatment resistant macular detachment and severe visual impairment in 6 eyes (4 patients). At the last visit, eyes with macular involvement were more common to had worse final best corrected visual acuity (≤20/50) than those without (9/12 vs. 0/8, *p* = 0.001).

**Conclusions:**

SRF usually develop early in the disease course in VKH patients who are not adequately controlled; it usually undergoes a highly dynamic process within the subretinal space and may involve the macula and resulted in poor final visual outcome.

## Background

Vogt-Koyanagi-Harada disease (VKH) is a multisystemic disease characterized by bilateral uveitis and frequently accompanying extraocular manifestations such as meningismus, vitiligo, alopecia, poliosis and dysacausia [1]. It has a genetic predisposition and primarily affects Asian, Middle Eastern, Hispanic and native American populations [2, 3], and is one of the most common panuveitis entities in the Chinese population [4–6]. The visual prognosis of VKH is generally good with prompt diagnosis and treatment, however, long-term uncontrolled and/or recurrence of inflammation may result in ocular morbidity by causing vision-threatening complications including cataract, glaucoma, choroidal neovascularization and subretinal fibrosis (SRF) [7].

Since firstly described in 1995, SRF has been well recognized as a sequela of chronic inflammation in VKH [7–12]. However, we previously reported 2 VKH cases (4 eyes) who developed SRF at acute uveitic stage within the second month of disease course [13]. Since then, 8 additional (altogether 10) VKH patients referred to our center at acute uveitic stage have developed SRF under close observation. The aim of the current study is to provide longitudinal observations of SRF in VKH, as well as to characterize the clinical features and outcomes of VKH patients with this finding.

## Methods

All VKH cases referred to our group between January 2008 and September 2015 at acute uveitic stage were reviewed retrospectively. Patients with SRF at presentation or developed SRF during follow up were included in this study. Diagnosis was made according to American Uveitis Society’s revised international criteria [14] and the following criteria, which is similar to previously described [13, 15], were used to define the acute uveitic stage: 1) disease duration at presentation should be less than 3 months; 2) the presence of at least 2 of the following signs that indicative of active posterior uveitis: optic disc edema, retinal edema, exudative retinal detachment, multifocal pinpoint leakage and/or subretinal dye pooling on fundus fluorescence angiography (FFA); and 3) the absence of vitiligo, alopecia, poliosis and depigmentation of the retinal pigment epithelium which are features of convalescent or chronic recurrent stages. A standard FFA was obtained at the first visit with TRC-50DX (Topcon Inc., Tokyo, Japan) or Spectralis HRA + OCT (Heidelberg Engineering, Heidelberg, Germany) unless the patient came with a printed report with good quality from other hospitals. Optical coherence tomography (OCT) scans using three-dimensional OCT 1000 (Topcon Inc., Tokyo, Japan) or Spectralis HRA + OCT with a 3-dimensional or a raster scan protocol centered on the fovea were obtained at presentation and follow up visits. The treatment regimen for VKH patients has been described previously [15]. In brief, an oral dose of 60-80 mg/d (1 mg/kg/d) prednisone or equivalent with gradual tapering was the mainstay and immunomodulatory treatment (IMT, mono- or combined treatment of cyclosporin A (CsA), cyclophosphamide (CTX) and azathioprine (AZA)) was indicated in severe, recurrent, and glucocorticoids under-responsive, dependent and intolerant cases. Cataract extraction with intraocular lens (IOL) implantation was indicated when substantial loss of vision was caused by a cataract and the anterior chamber had been quiescent (≤0.5 + cells according to the Standardization of Uveitis Nomenclature criteria [16]) for a minimum of 3 months with no more than 20 mg/d of oral prednisone or equivalent. Trans pars plana vitrectomy (PPV) and removal of SRF was performed in patients with persistent macular detachment due to SRF and were willing to receive the surgery.

Statistical analyses were processed by SPSS 22.0 for Windows (SPSS Inc.). Two-sided Fisher exact test were used for statistical analysis and a *p* value less than 0.05 was considered statistically significant.

## Results

Of 193 cases reviewed, 10 patients (6 males and 4 females) with a median age of 39.0 (range, 23 to 58) years old were included. Baseline demographics, clinical characteristics, treatments and final visual outcomes of the patients are presented in Table 1. The median disease duration at presentation and median duration of follow up were 25.5 (range 5 to 60) days and 32.5 (range 13 to 61) months respectively (Table 1). At presentation, all except patient 1 had been inappropriately treated with glucocorticosteroid (insufficiently dosed or tapered too fast) for longer than 2 weeks (Table 1). Despite that all patients received large dose glucocorticosteroid (1 mg/kg/d prednisone or equivalent) and IMT agents at their therapeutic dosages immediately after presentation, 10 (100%), 7 (70%), 5 (50%) and 7 (70%) patients had recurrent anterior uveitis (RAU), macula edema (ME), macular atrophy (MA) and cataract/IOL in at least one eye during follow up, respectively (Table 1). Compared to baseline, all eyes had better BCVA at the last visit and the medium logarithm of the minimum angle of resolution (logMAR) best corrected visual acuity (BCVA) improved from 1.76 (range: 3.0 to 0.60) and 1.52 (range: 3.0 to 0.70) at presentation to 0.41 (range: 1.70 to −0.08) and 0.049 (range: 1.0 to −0.08) at the final visit in the right and left eyes, respectively.

**Table 1:** Baseline demographics, clinical characteristics and treatments of the VKH patients with subretinal fibrosis

Pt No.	G	Age	DFV(d)	Inappropriate initial CS treatment>2w^a^	IBCV (OD)	IBCV (OS)	IMTs and initial daily doses	RAU	ME	MA	Cataract/ IOL	MI	SRFRS	FBCV (OD)	FBCV (OS)	DFU (m)
1	M	23	5	–	20/80	20/100	CsA (200 mg/d) + AZA (100 mg/d)	+	+	–	+	OS	–	20/25	20/80	40
2	M	28	22	Insufficient dosing	20/2000	20/2000	CsA (200 mg/d)	+	+	–	+	OD	–	20/1000	20/16	24
3	M	52	27	Over-rapid tapering	20/100	20/330	CTX (100 mg/d) /CsA (200 mg/d)	+	+	+	+	OU	–	20/33	20/16	43
4	F	50	20	Insufficient dosing	20/2000	20/400	CTX (100 mg/d)	+	+	–	+	OU	–	20/133	20/100	61
5	M	42	60	Over-rapid tapering	20/660	20/400	CsA (200 mg/d)	+	+	+	+	OU	OD	20/200	20/200	25
6	M	31	28	Over-rapid tapering	20/2000	20/2000	CsA (200 mg/d)	+	–	+	+	OU	OU	20/250	20/50	42
7	F	55	37	Over-rapid tapering	20/100	20/660	CsA (150 mg/d)	+	+	+	–	OU	OS	20/80	20/100	57
8	F	58	20	Insufficient dosing	20/660	20/660	CsA (150 mg/d)	+	+	+	+	–	–	20/25	20/25	24
9	F	36	35	Over-rapid tapering	20/2000	20/2000	CsA (150 mg/d)	+	–	–	–	–	–	20/20	20/20	24
10	M	25	50	Over-rapid tapering	20/2000	20/2000	CsA (200 mg/d)	+	–	–	–	–	–	20/16	20/20	13

*Abbreviations: Pt* patient, *G* gender, *F* female, *M* male, *DFV* duration at first visit, *CS* corticosteroid treatment, *IMT* immunomodulatory treatment, *CsA* cyclosporin A, *CTX* cyclophosphamide, *AZA* azathioprine, *IBCV* initial best corrected visual acuity, *RAU* recurrent anterior uveitis, *ME* macular edema, *MA* macular atrophy, *IOL* intraocular lens, *MI* macular involvement, *SRFRS* SRF removal surgery, *FBCV* final best corrected visual acuity

“+” denotes positive for the indicated finding/surgery in at least one eye while “-” denotes negative

^a^: inappropriate initial corticosteroid treatment for longer than 2 weeks before being presented to our center

**Fig. 1:**
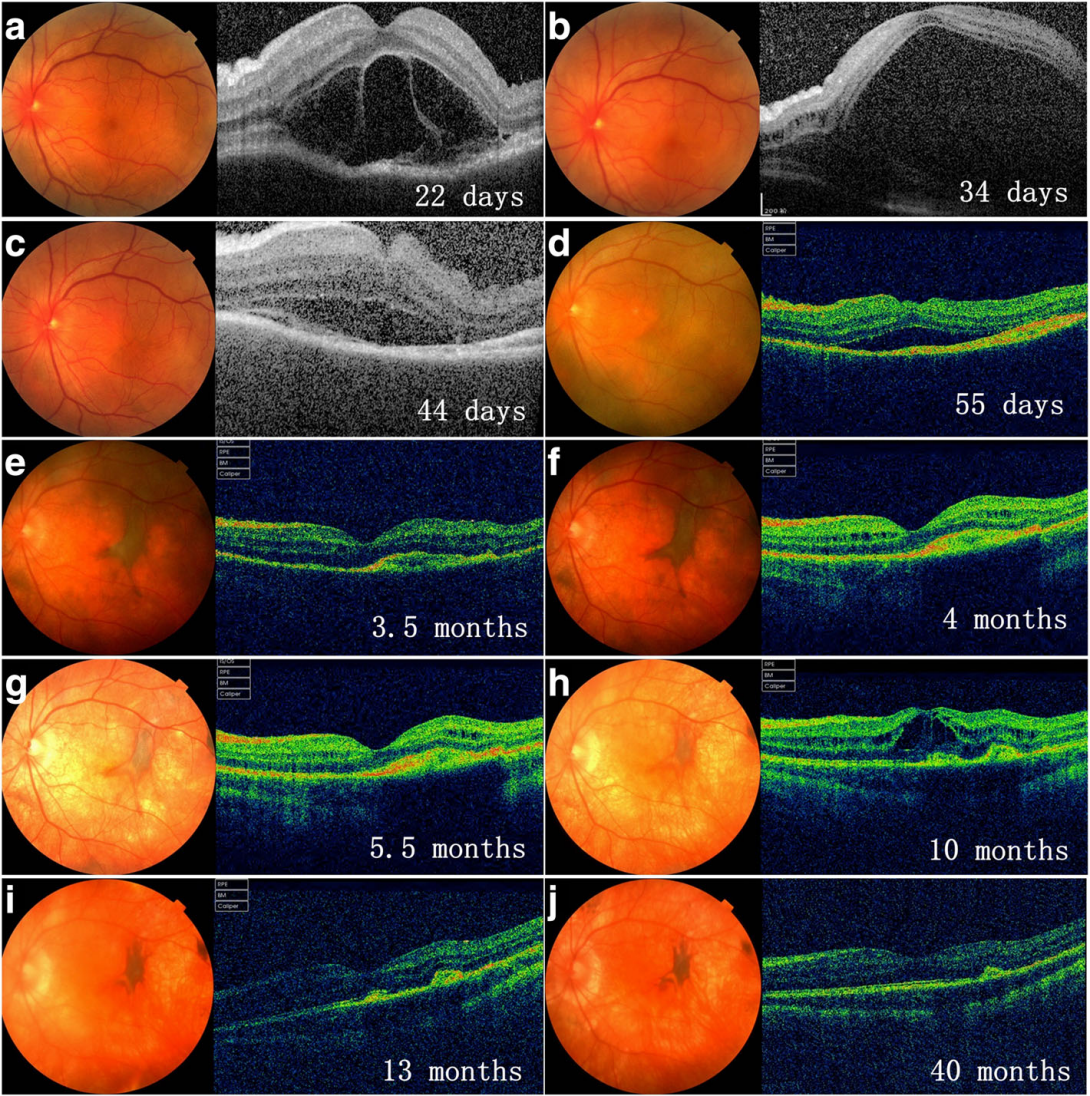
Longitudinal documentation of optical coherence tomography (OCT) and fundus photography: patient 1. Subretinal fibrosis (SRF) was barely detectable (**a**, **b**) until at day 44 of disease course (**c**), which appear as a pigmented subretinal membrane on fundus photograph and a hyper-reflective layer above the retinal pigment epithelium (RPE) on OCT (**c**). SRF then underwent shape-change/migration and proliferation/pigmentation under the subretinal space (**d**, **e**), and then stabilized (**f**). De-pigmentation of SRF along with the whole fundus ensued thereafter (**g**, **h**), followed by re-pigmentation of SRF (**i**, **j**). Macular edema was detected at 10 months of disease course (**h**) and responded well to sub-conjunctival injection of triamcinolone acetonide

**Fig. 2:**
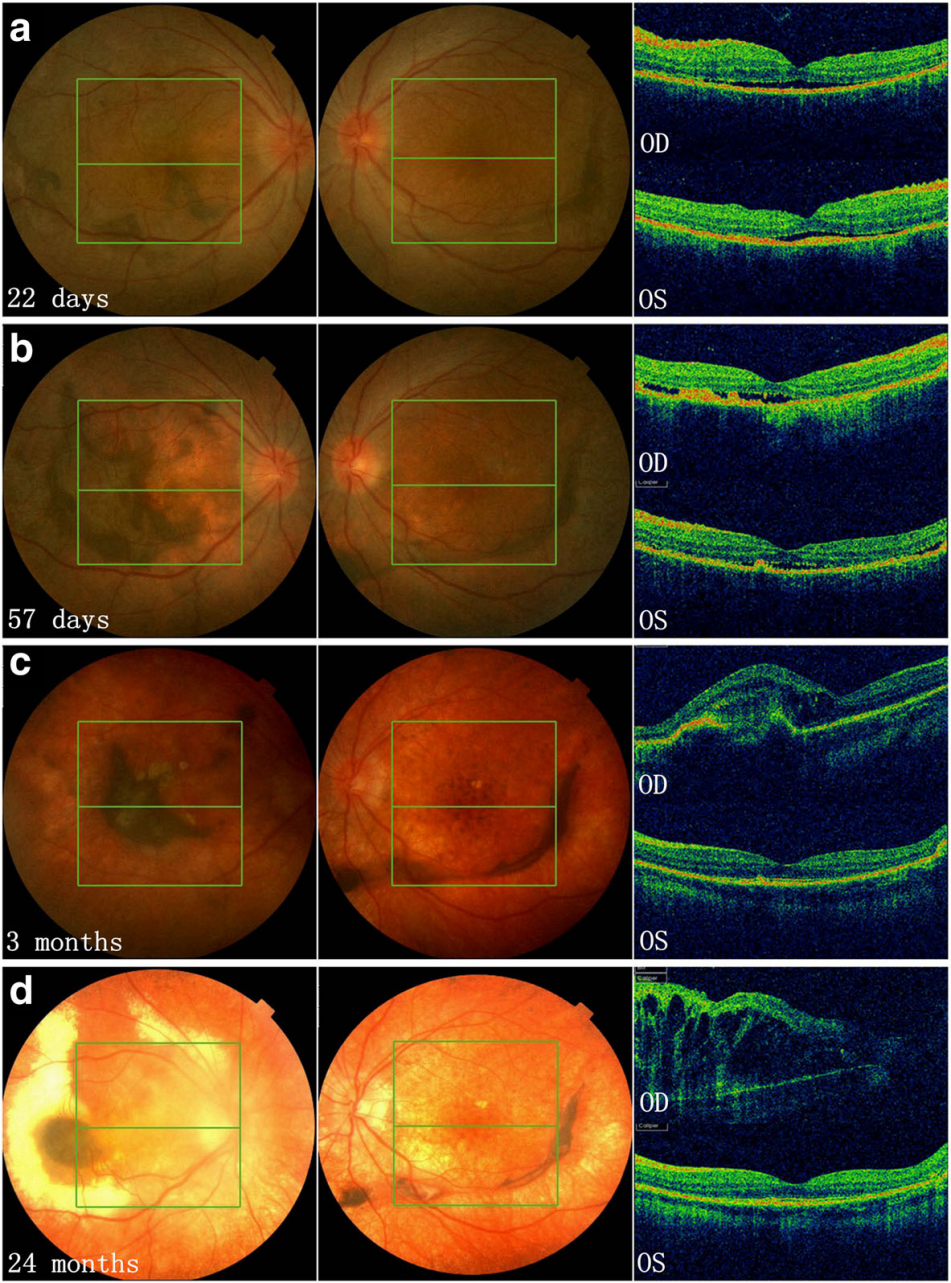
Longitudinal documentation of optical coherence tomography (OCT) and fundus photography: patient 2. Subretinal fibrosis (SRF) was present in both eyes at the first visit on day 22 of disease course (**a**), which then underwent shape-change/migration and proliferation/pigmentation until 3 months after disease onset (**b**, **c**). Depigmentation of SRF along with the fundus was observed in both eyes thereafter; while the SRF in the left eye remain quiescent in bulk and shape, that in the right eye continued to change slowly in location and shape, and finally involved the whole macula at the last visit with profound macular edema (**d**) and a best corrected visual acuity of 20/1000 (comparing to 20/16 in the left eye)

**Fig. 3:**
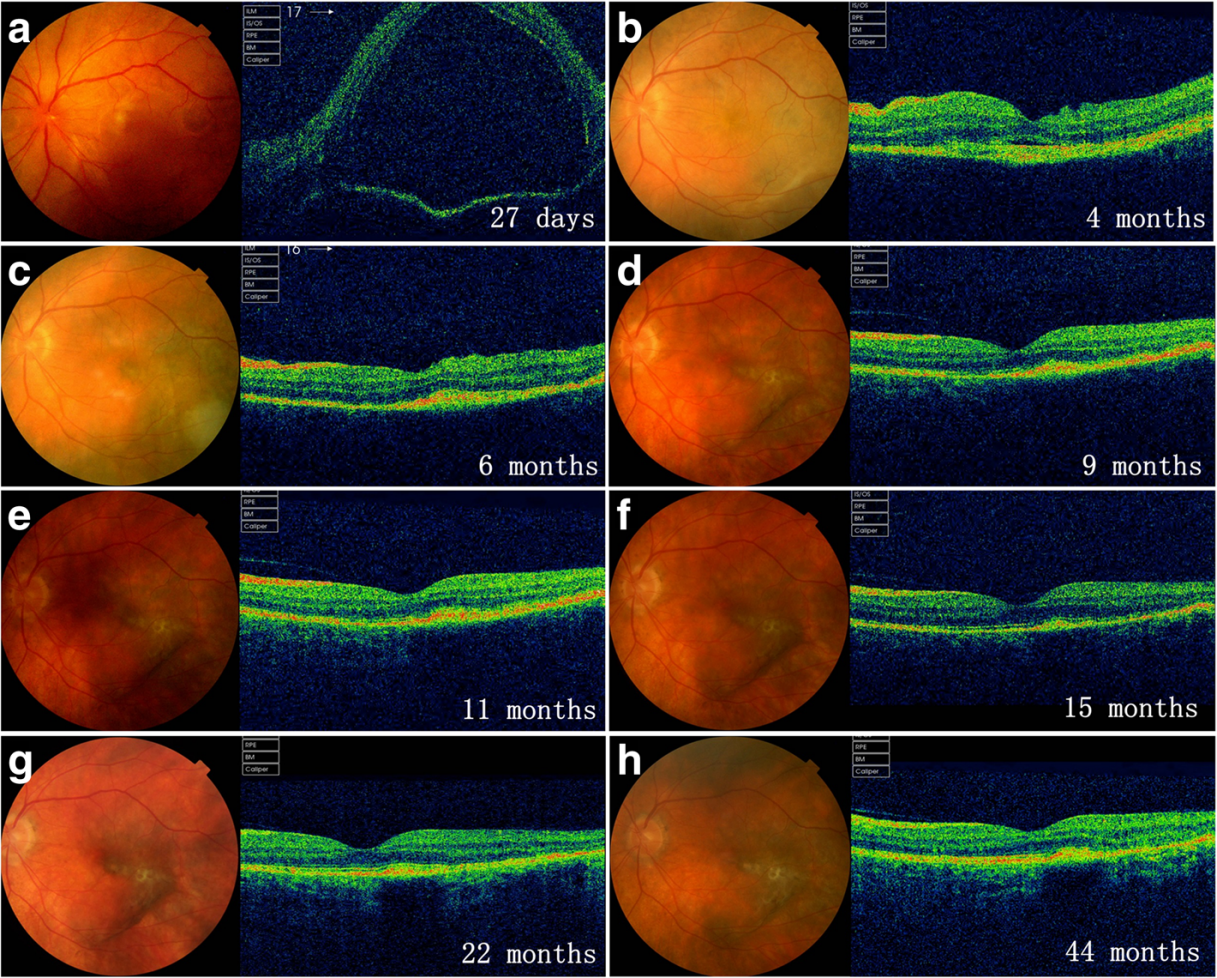
Longitudinal documentation of optical coherence tomography (OCT) and fundus photography: patient 3. At presentation (27 days after onset), OCT revealed no subretinal exudates (SRE) but bullous retinal detachment (**a**). The patient was then lost to follow up until 4 months after onset when subretinal fibrosis (SRF) was documented (**b**). Shape-change/migration and proliferation/pigmentation of SRF was observed thereafter until it reached quiescence at 9 months of disease course (**c** to **h**)

**Fig. 4:**
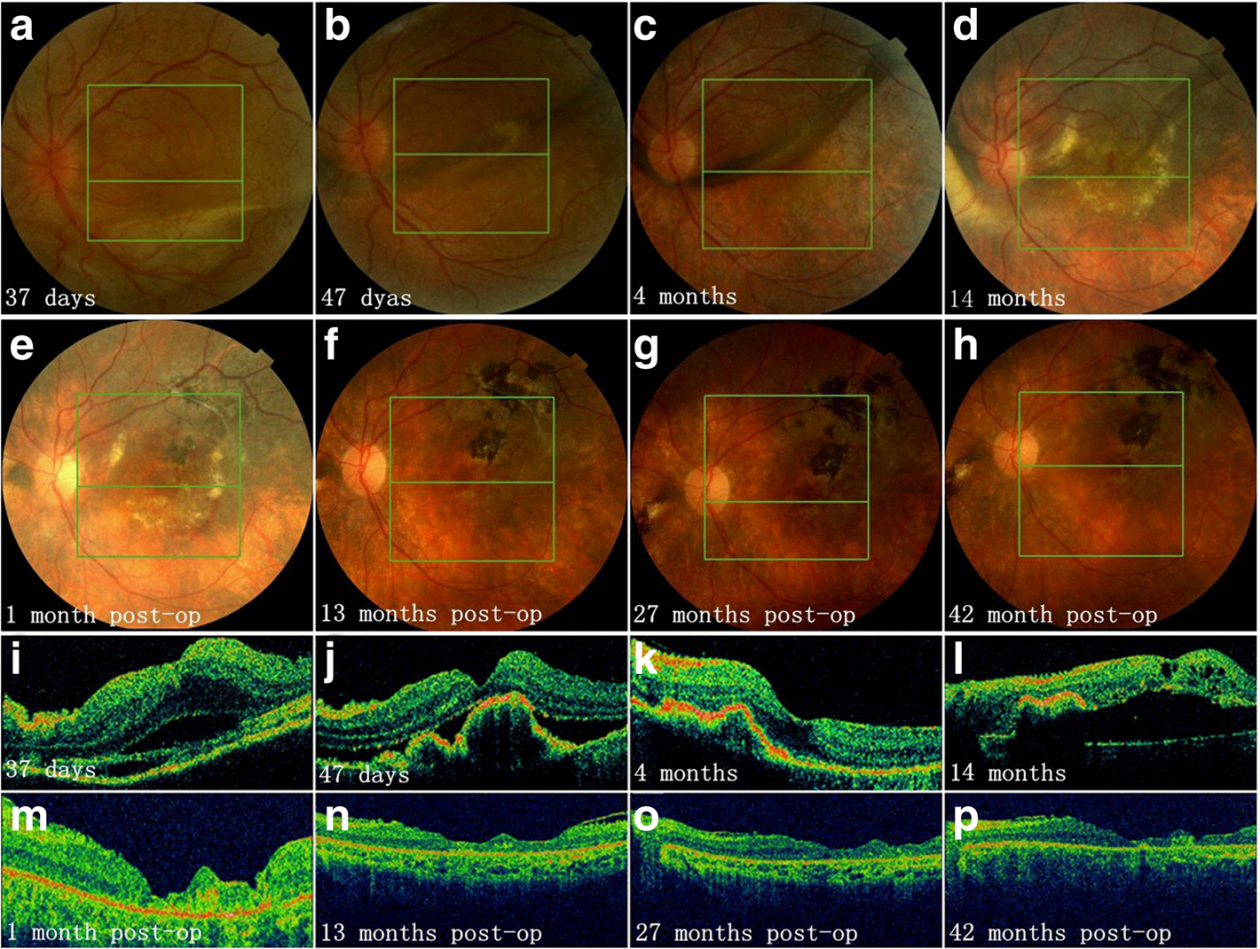
Longitudinal documentation of optical coherence tomography (OCT) and fundus photography: patient 7. Fundus photographs from day 37 to 4 months after onset revealed shape-change/migration and proliferation/pigmentation subretinal fibrosis (SRF) (**a** to **c** and **i** to **k**). Vitrectomy and SRF removal was performed 15 months after disease onset due to macular detachment caused by SRF (**d** and **l**). Complete absorption of subretinal fluid was observed after surgery (**e** to **h**, **m** to **p**) with best corrected visual acuity improved from 20/250 (before surgery) to 20/100

**Fig. 5:**
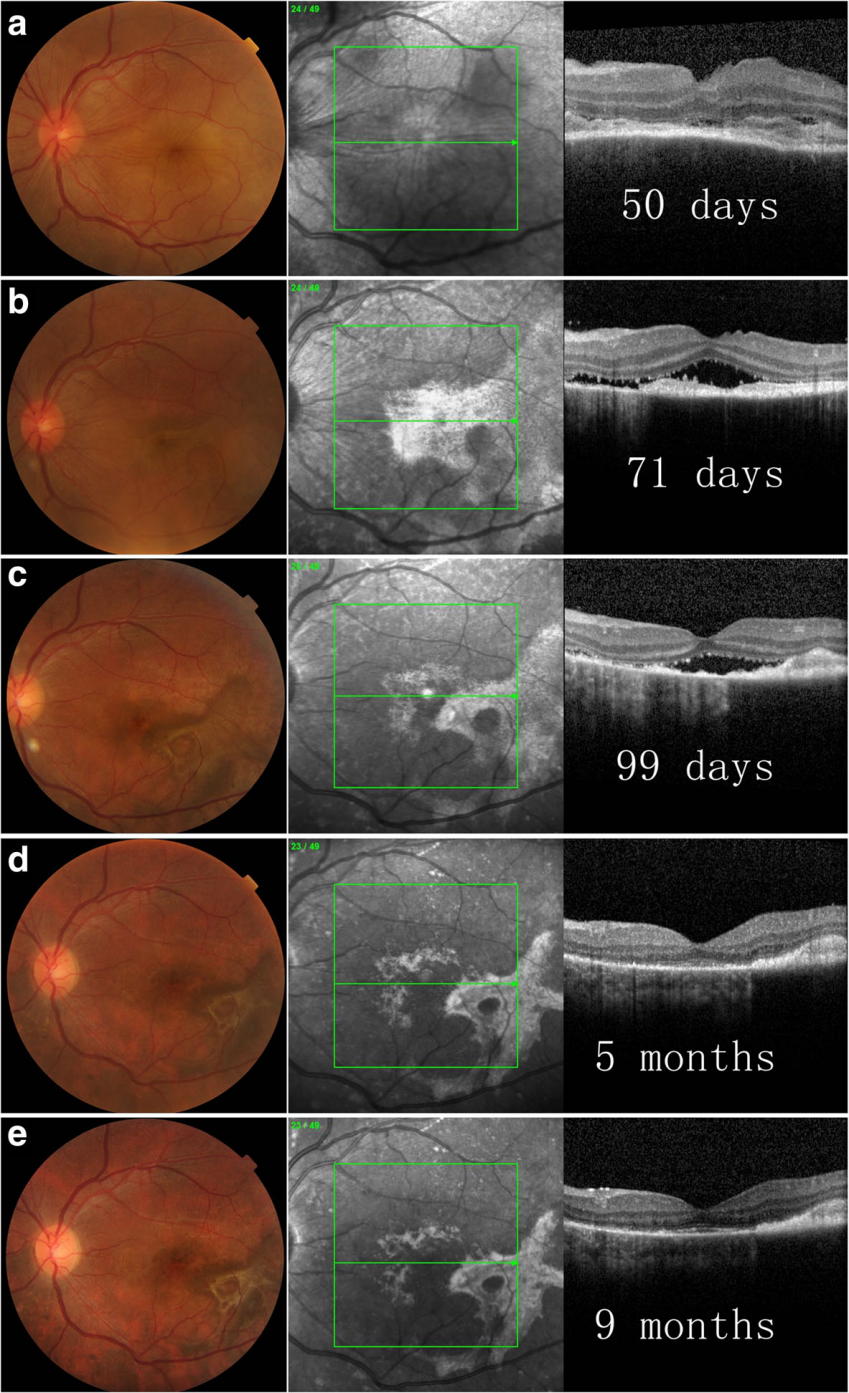
Longitudinal documentation of optical coherence tomography (OCT) and fundus photography: patient 10. At day 50 (**a**), subretinal fibrosis (SRF) was not observed in color and infrared (IR) fundus photographs, and OCT scan revealed a layer of hypo- to medium- reflectivity above the retinal pigment epithelium (RPE), which might be subretinal exudate. SRF was observed in both color and IR fundus photographs at day 71 (**b**), which then underwent shape-change/migration and proliferation/pigmentation (**c**, **d**), and then stabilized (**e**)

All patients developed bilateral SRF within the first 4 months of disease course and 7 (70%) patients within the first 2 months. In 8 (80%) patients, shape-change/migration and progressive proliferation/pigmentation of SRF was observed over a period of several months after its formation (Figs. 1, 2, 3, 4 and 5), and then became quiescent but may further underwent depigmentation or pigmentation (Figs. 1 and 2).

SRF involved macula in 12 eyes (7 patients) and caused treatment resistant macular detachment and severe visual impairment in 6 eyes (4 patients) (Figs. 2 and 4), of which 4 eyes (3 patients) underwent and benefited from surgical removal of SRF (Table 1, Fig. 4). At the last visit, eyes with macular involvement were more common to had worse final BCVA (≤20/50) than those without (9/12 (75%) vs. 0/8 (0%), *p* = 0.001).

## Discussion

SRF was first noted in VKH patients in 1995 and described more comprehensively later by the same group [8] and another [9] as a complication of chronic disease. Up to date, it has been reported by a number of studies in VKH patients of diverse geographic and ethnic origins [7, 10–12, 17, 18]. In VKH patients from Southern and Eastern Asia, SRF appeared to be quite uncommon, as it was either not noted [19–22] or observed only in less than 2% of patients when present [12, 18].

Lertsumitkul et al. [8] characterized for the first time the following important features of SRF in VKH patients: 1) SRF tend to appear in patients with longer duration of disease and more severe ocular inflammation, 2) involve more frequently areas within the retinal arcades and proximal to the optic disc, and 3) is an independent risk factor of poor visual prognosis. Kuo et al. [9] described some additional characteristics of SRF in VKH including its tendency to involve both eyes, its association with exudative retinal detachment and multiple recurrences of either anterior or posterior uveitis, and its potential to cause severe visual impairment when involve the macula. In accordance with the above mentioned pioneer studies, patients in our study all had bilateral involvement, had high rates of complications from the anterior segment to the fundus (RAU, cataract, ME and MA) and poor final BCVA (≤20/50); SRF tended to involve the macula and might further result in treatment resistant macular detachment which warranted surgery. Our experiences on surgical treatment of SRF in VKH patients are beyond the scope of the current study and have been published elsewhere [23].

SRF typically form in areas with intense choroidal inflammation where the choroid is thickened due to infiltration of inflammatory cells, or where the retina is detached with subretinal inflammatory exudates rich in leukocytes and fibrin [24]. The mechanism of formation of SRF is supposed to be a type 2 epithelial-mesenchymal transition involving interactions of resident cells (retinal pigment epithelium (RPE) cells in this context) with infiltrated inflammatory cells and their products (fibrin, a variety of pro-inflammatory and fibrogenic cytokines) [8, 25, 26]. The following observations of SRF in our study were in accordance with the mechanism in that 1) SRF usually develops in acute uveitis stage in patients who were not adequately controlled, suggesting that severe inflammation might be a prerequisite for development of SRF; and 2) the shape-change/migration and proliferation/pigmentation process observed in most of our patients suggests participation of local cells (RPE etc.).

On the other hand, more aggressive immunosuppressive treatments, such as intravenous corticosteroid pulse therapy, might be needed for patients with early SRF since oral corticosteroid combining IMT were not observed to be sufficient to control further development of SRF and to prevent various complications in most of our patients. It is also reasonable that early intense treatment may prevent formation of SRF by promoting rapid resolution of choroidal inflammation. Evidences are accumulating that early combination of IMT with corticosteroid may improve prognosis in VKH patients [27–30], although contradictory results have also been reported [20]. In particular, Abu EAM and colleagues recently reported that mycophenolate mofetil (MMF) in combination with systemic corticosteroids as first-line therapy prevents progression of acute VKH toward chronic recurrent phase and development of sunset glow fundus [27].

It is worth mention that choroidal neovascularization (CNV) has similar anatomical location and funduscopic appearance to SRF and was reported to be more prevalent than SRF in VKH in the two pioneer studies [7, 8]. Notably, in Lertsumitkul et al.’s study, co-presence of CNV and SRF was found in 7 out of 75 VKH patients [8]. However, the differences between these two findings have not been adequately discussed. While FFA and Indocyanine green angiography (ICGA) is essential to establish the diagnosis of CNV [8], the following features of SRF observed in this study may also be useful to make a right differential diagnosis between CNV and SRF: SRF usually develop at acute uveitic stage, is larger in size and less regular in shape, and usually undergo shape-change/migration and progressive pigmentation over a period of several months after formation.

Our current study, however, is limited by its retrospective nature and limited number of patients. The disease durations of the patients might also be subjected to potential recall bias. In addition, multimodality imaging approach usually provide better perception and more comprehensive understanding of complex pathological fundus findings such as SRF. ICGA, enhanced depth imaging OCT (EDI-OCT) are useful techniques to detect early and subclinical choroidal inflammation in VKH; fundus autofluorescence (FAF) provides a topographic map of the distribution of lipofuscin and, therefore, is an important imaging modality for observation of RPE associated pathology. Future prospective studies are needed to confirm our findings and should adopt multiple imaging modalities.

## Conclusions

Our observations reveal that in VKH patients, SRF usually develops at acute uveitic stage and undergoes gradual shape-change/migration and progressive proliferation/pigmentation over a period of several months after formation; SRF is associated with high risks of various complications (RAU, cataract, ME and MA) and poor visual acuity (≤20/50), and might cause refractory macular detachment with severe visual impairment when involve the macula.

## Abbreviations

AZA: Azathioprine
BCVA: Best corrected visual acuity
CNV: Choroidal neovascularization
CsA: Cyclosporin A
CTX: Cyclophosphamide
EDI-OCT: Enhanced depth imaging OCT
FAF: Fundus autofluorescence
FFA: Fundus fluorescence angiography
ICGA: Indocyanine green angiography
IMT: Immunomodulatory treatment
IOL: Intraocular lens
logMAR: Logarithm of the minimum angle of resolution
MA: Macular atrophy
ME: Macular edema
MMF: Mycophenolate mofetil
OCT: Optical coherence tomography
PPV: Trans pars plana vitrectomy
RAU: Recurrent anterior uveitis
RPE: Retinal pigment epithelium
SRF: Subretinal fibrosis (SRF)
VKH: Vogt-Koyanagi-Harada disease
